# Supplementary material for: A set of multi-entry identification keys to African frugivorous flies (Diptera, Tephritidae)
Source: Zookeys. 2014 Jul 24;(428):97–108. doi: 10.3897/zookeys.428.7366 (PMC4143993; doi:10.3897/zookeys.428.7366)
Supplement: Supplementary material 6 — Key to Ceratitis [file zookeys-428-097-s006.zip › SF6_ZooKeys_key to Ceratitis/key/SF6_ZooKeys_key to Ceratitis/Media/Html/Ceratitis lunata .htm]

Ceratitis (Ceratalaspis) lunata Munro


***Ceratitis*** **(*Ceratalaspis*)
*lunata* Munro**

*Ceratitis* (*Pardalaspis*) *lunata* Munro, 1935: 305.

Body
length: 4.49 (3.85-5.00) mm; wing length: 4.65 (4.40-4.90) mm.

Male

Head.
Antenna yellow-orange. Third antennal segment slightly more than twice as long
as second segment. Arista distinctly plumose. Frons with short scattered hairs
which are largely the same colour as frons, sometimes slightly darker; convex,
in lateral view not distinctly projecting forwards at antennal implant.
Chaetotaxy normal for subgenus.

Thorax.
Postpronotum white, with relatively small black spot. Ground colour of
mesonotum pale with orange tinge; mesonotal pattern, prescutellar spots not
touching intra-alar spots, median sutural spot missing. Chaetotaxy normal for
subgenus. Inner scapular setae pale, outer ones black. One anepisternal
bristle. Scutellum white basally, otherwise yellow with three black separate
markings apically; basally with two dark spots. Subscutellum pale with three
dark separate spots, except along dorsal margin where touching.

Legs
yellow; setation typical for subgenus, mainly pale especially on femora.
Posterior and posterodorsal rows on fore femur pale.

Wing
bands with markings extensively yellow. Banding, marginal band continuous;
cubital band free; medial band absent; crossvein r-m at middle of discal cell.
Crossvein dm-cu posteriorly more inwards than anteriorly.

Abdomen.
More or less brownish. Setation and banding typical for subgenus.

Female.

As
male except for the following characters: arista with slightly shorter hairs.
Postpronotal spot larger. Outer scapular seta (only present in paralectotype)
reddish (inner missing in all specimens). Subscutellum completely dark. Wing
with crossvein dm-cu posteriorly more outwards than anteriorly. Abdomen yellow
in paratype. Oviscape shorter than abdominal terga 3-6 combined.

(Description
after De Meyer, 1998)
